# Women’s voices, emotion and empathy: engaging different publics with ‘everyday’ health histories

**DOI:** 10.1136/medhum-2020-012102

**Published:** 2021-05-25

**Authors:** Tracey Loughran, Kate Mahoney, Daisy Payling

**Affiliations:** History, University of Essex, Colchester, Essex, UK

**Keywords:** medical humanities, cultural history

## Abstract

This article explores our experiences on a Wellcome Trust-funded project on women’s experiences of ‘everyday health’ in Britain between the 1960s and the 1990s. We explore issues around researching ‘everyday health’, including the generation and interpretation of source materials, and the role of empathy and emotion in interactions with different audiences as we share these materials in public engagement activities. We discuss three case studies of engagement activities to draw out potential uses of source materials and the responses of different audiences to these materials, and reflect on what we have learnt since embarking on these public engagement activities. We took into our interactions with different audiences the belief that fully historicised understandings of ‘health’ enrich individual lives and create new capacities for meaningful action now. The public engagement activities we carried out reinforced this belief, but also caused us to question some of our assumptions. In particular, an activity with trainee healthcare professionals designed to demonstrate how active and empathetic listening can prevent the unintentional infliction of harm in healthcare settings achieved this end—but did so in a way that was itself unintentionally insensitive to the pressures healthcare professionals face. Medical humanities can help to contextualise, nuance and improve healthcare practice—but only through active listening and dialogue across medicine and the humanities. We conclude by considering how these activities, which currently rely on the interpersonal relations of the team with audiences, might be adapted and preserved in digital form beyond the span of the project.

## Introduction

We are a team of historians working on the project ‘Body, Self, and Family: Women’s Emotional, Psychological, and Bodily Health in Britain, c. 1960–1990’ (hereafter BSF).1 This project aims to create an intersectional history of women’s ‘everyday health’ from the ground-up. We examine how sweeping social changes affected women’s embodied and emotional subjectivities at different stages of the life cycle; the importance of ‘race’, sexuality and social class in differentiating women’s experiences of health, illness and well-being; and how women negotiated different sources of expertise and authority. The project has generated multiple public engagement activities. These activities use materials created and located in the course of the research, including oral histories and content from mass-market women’s magazines, to historicise understandings of gender, embodiment, and well-being and encourage audiences to think differently about their capacities for action in the present.

In this article, we explore issues around researching ‘everyday health’, including the generation and interpretation of source materials, and the role of empathy and emotion in interactions with different audiences as we share these materials in public engagement activities. We discuss three case studies of engagement activities to draw out potential uses of source materials and the responses of different audiences to these materials, and reflect on what we have learnt since embarking on these public engagement activities. We took into our interactions with different audiences the belief that fully historicised understandings of ‘health’ enrich individual lives and create new capacities for meaningful action now. The public engagement activities we carried out reinforced this belief, but also caused us to question some of our assumptions. In particular, an activity with trainee healthcare professionals designed to demonstrate how active and empathetic listening can prevent the unintentional infliction of harm in healthcare settings achieved this end—but did so in a way that was itself unintentionally insensitive to the pressures healthcare professionals face. Medical humanities can help to contextualise, nuance and improve healthcare practice—but only through active listening and dialogue across medicine *and* the humanities. We conclude by considering how these activities, which currently rely on the interpersonal relations of the team with audiences, might be adapted and preserved in digital form beyond the span of the project.

## Researching ‘everyday health’

The Body, Self and Family project researches ‘everyday health’, meaning the emotional, psychological and bodily state-of-being in individuals’ day-to-day lives, and the strategies they pursue (or do not) to maintain equilibrium in this state-of-being. This concept of ‘everyday health’ is broad and can be applied to every aspect of embodied experience, meaning that it is also highly adaptable to different contexts of healthcare—a point that is particularly important in considering how different audiences, and perhaps above all healthcare practitioners, might engage with the findings of this project. In settings where hospital-based medicine is often the focus, thinking about ‘everyday health’ can encourage a more holistic and/or public health-oriented outlook. In this connection, Stephen Hinchcliffe *et al*’s concept of ‘healthy publics’ as ‘dynamic collectives of people, ideas and environments that can enable health and well-being’ is especially fruitful in capturing the diversity and fluidity of health behaviours and contexts incorporated in our research and public engagement activities.2 On the project, we use many different source materials to capture the diversity of ‘everyday’ experiences, negotiate the complex relationships between representation and experience, and grapple with the problem of how political and social structures shape the capacity for agency. Oral history and mass-market women’s magazines are particularly important sources for both our research and public engagement activities.

The project will generate 50 oral history interviews with women of diverse ethnic backgrounds and sexual orientations born between 1940 and 1970 from across the UK (so far, we have completed nearly half of these interviews). Interviews are semistructured around the ‘life story’ approach, moving through sections on ‘childhood and growing up’, ‘adolescence, adult life, and relationships’, ‘fertility, family life, and other health experiences’ and ‘reflections on growing older’. Direct questions about health experiences and medical encounters are located within the wider context of the interviewee’s life story in order to illuminate her day-to-day emotional, psychological and bodily state-of-being in the past—all the mundane aspects of experience that are less easily recalled than more dramatic incidents and encounters. We draw on the concept of ‘composure’ to understand how interviewees make sense of their bodily, psychological and emotional experiences, construct coherent life stories through the act of narration, and negotiate between public and private ‘scripts’ in telling their stories.3

We also make extensive use of mass-market women’s magazines, including both ‘service’ magazines aimed at women in the home, and magazines that catered for ‘liberated’ women.4 In the early 1960s, 50.2 million women in Britain read a weekly magazine, and 34 million read a monthly. By 1987, the figure for weekly magazines had declined to 23.9 million, while that for monthlies had risen to 40 million.5 These publications therefore had considerable reach throughout our period. In their appeal to women as consumers and caregivers, they carried extensive health-related content, and so help us to chart those ideas about health and illness that formed the backdrop of ‘ordinary’ life (itself a concept that gained increasing cultural and political purchase in the postwar period).6 This content appeared across multiple formats (features, first-person accounts, interviews, readers’ letters, ‘expert’ columns and advertising), produced by named intermediaries including feature writers, columnists, doctors and agony aunts. From the early 1960s, magazines also started to introduce more interactive and reader-generated content, as part of the attempt to build readers’ loyalty.7 Oral history and mass-market magazines are therefore ‘ground-up’ sources that help us understand the thoughts, feelings and horizons of knowledge of women who would not otherwise enter the historical record.

In practice, accessing diverse experiences of ‘everyday health’ is not quite that simple. There are always gaps. For example, our initial recruitment appeal yielded no BAME or LGBTQ+ oral history interviewees. Our next recruitment phase targeted BAME (Black and Minority Ethnic) and LGBTQ+ (Lesbian, Gay, Bisexual, Transgender, Queer, +) groups and events. This meant that some of participants entered the interview aware that they were speaking *as* BAME or LGBTQ+ women—a burden of representation that did not fall on our white, heterosexual participants. In addition, mass-market women’s magazines were not diverse or inclusive in our period. They assumed the white, heterosexual woman as the standard reader, rarely depicted non-white faces until the 1980s, and when they did present the perspectives of BAME or LGBTQ+ women, framed this as a self-conscious act of inclusion. There were no British mass-market magazines for BAME or LGBTQ+ women until the mid-1980s.8 As historians, we need to add more diverse groups of women to the picture, but also portray the *lack* of representation in the past, and how this affected women’s lives.

These sources also pose multiple problems of interpretation. It is never easy to read subjectivity from discourse.9 We do not access women’s voices directly in mass-market magazines: reader content was carefully selected and heavily edited, and while it is the best available evidence of what certain readers thought and felt, it requires delicate handling. These magazines also carry hefty ideological freight. Early feminist scholarship argued that women’s magazines fostered false consciousness, entrenched capitalism and reconciled women to their narrow lives.10 Since the 1980s, scholarship has emphasised that magazines are a heteroglossic form that readers actively negotiate—but *how* readers enact this agency, especially in relation to past reading experiences, remains hazy.11 Likewise, oral history’s living link to the past is always highly mediated. Memory fades and is reworked over time, as individuals seek to understand past events in the changed circumstances of their own lives, and as the interpretive frameworks within the wider culture shift. Moreover, the interview process itself shapes how interviewees talk about their experiences, as well as what they recall: the intersubjective relationship of interviewer and interviewee, questions and interview environment all contribute to what is said and what is left unspoken, unarticulated or unremembered.12 The past is not just ‘out there’, waiting to be recovered.

## Empathy, emotion and ‘everyday health’ in public engagement

In our public engagement activities, we use oral histories and mass-market magazines in the aim of fostering new understandings of gender, embodiment, and ‘everyday health’ among different audiences. In prompting audiences to consider continuities and changes in these areas over time, we want to challenge unreflective assumptions about linear progress, and open up the radical contingency of past and present. Awareness of unfamiliar pasts prises open the possibility of different futures, via participants’ new understandings of their own potential to act in the present. History is particularly good at demonstrating the power of structural forces, and therefore helping individuals recognise how and where to direct their action effectively.13 Our aim is therefore to use history to empower audiences.

We are not prescriptive about the forms such action might take. Audiences bring their own experiences to bear on the activities we have designed, and we cannot determine their understandings of these materials or predict what they will do next. For us, the important point is to offer audiences different perspectives, and to foster a dynamic relationship with historical materials—but that interaction is only partially determined by our design and conduct of the activities, and indeed only partially knowable by us. In encouraging audiences to think differently about their capacities for action in the present, we do not assume that we know best, or that our ideal or projected outcomes are the only possible. Likewise, while we have not adopted a co-production model, we always attempt to remain open to what we might learn from audiences, and to continuously reflect on and adapt activities in response to our interactions with different publics. This is a dialogical model in the sense that we value both the implied meaning of the activities (as we see it) and the interpretations of audiences (their perspectives as well as our own).

In our view, empathy is a crucial, but always complicated, aspect of this dialogue. Empathy, broadly defined as ‘the act of coming to experience the world as you think someone else does’, is crucial to achieving this aim of empowering audiences.14 Most often, public engagement audiences initially respond empathetically to our source materials without much prompt or direction. Our area of research is relatable, perhaps especially to girls and women, as it is a project on women’s history. However, everyone is a gendered being, everyone inhabits a body, and everyone feels ill or well at different times. Similarly, the materials we use resonate with audiences: the first-person testimonies of oral histories encourage identification; magazines provoke pleasurable memories in those old enough to remember these publications, while formats are sufficiently recognisable to make sense to those young enough to be raised on social media. These resonances and identifications usually contain emotional elements, though the emotions stimulated vary according to individuals’ experiences and interpretations—nostalgia or regret for lost pasts, anger at perceived injustices or joy at recognition—and are not responses we could seek to control. While we aim to make the past and the present *less* familiar, this immediate empathetic (and emotional) response is necessary to hold audiences’ attention and then engage them at a deeper level.

It is commonplace to assume that the ability to emotionally inhabit the lives of others is necessarily good. However, the social value of empathy is contested. The psychologist Paul Bloom argues that empathy provides a poor guide to moral decision-making, with immediate, emotional responses tending to bias and limitation in contrast to ‘conscious, deliberative reasoning’.15 In debates on the history curriculum in England, from the 1980s conservative commentators caricatured the cultivation of empathy, as opposed to the inculcation of facts, as a preoccupation of loony lefties seeking to erode ‘our’ glorious island story.16 Specific reference to ‘historical empathy’ dropped off the national curriculum in 1997.17 The current curriculum requires study of ‘the diverse experiences and ideas, beliefs and attitudes of men, women and children in past societies and how these have shaped the world’, but there is no reference to empathy, identification or imagination in this context.18 These debates on historical empathy are not over, nor limited to discussion of the curriculum. As Christine Slobogin examines in her contribution to this special issue, negative characterisations of historical empathy are still prevalent today.

Advocates of historical empathy (ourselves included) believe there is no necessary opposition between empathy and reason. Rather, a properly *historicised* empathy depends on the contextualisation that precedes and follows from engagement with historical evidence—in other words, from the fusion of ‘facts’, interpretation and imagination.19 This is not an immediate or facile response. As Slobogin states, empathy ‘requires some work, some imagination’. Moreover, empathy is not only a tool to engage audiences, or to educate students. It is also an essential aspect of how historians engage with sources—how *we* respond to traces of the past. With the exception of oral history, where interaction with living participants forces attention to emotion, historians rarely explore their emotional and empathetic relations to source material.20

Speaking for ourselves, as oral historians we know it is not possible to listen to our interviewees without trying to inhabit their worlds. Our agreements and disagreements about these empathetic connections have generated some of our most productive discussions about how to understand our participants’ stories. As feminist scholars, we also know that the profusion of idealised images in magazines that we read for research affect us emotionally too. As women, we are the audience that is *supposed* to identify with as well as desire these visions of femininity. Our responses are not identical to the resonances of these images for the original readers, but nevertheless help us to understand what was at stake for those readers. Empathy is not a replacement for historical understanding. However, our empathetic responses are shaped by what we know about the past *as historians*, and in turn help to form the questions we ask about that past. As this suggests, we fully agree with Christine Slobogin’s spirited defence of the concept of historical empathy in her contribution to this special issue. A conscious, deliberate and informed historical empathy immeasurably enhances historical understanding.

We aim to nurture historical empathy as an active force that is an inextricable part of historical knowledge, in both our research and our public engagement activities. As Leslie Jamison asserts, empathy ‘isn’t just something that happens to us – a meteor shower of synapses firing across the brain – it’s also a choice we make: to pay attention, to extend ourselves’; a choice requiring ‘exertion, that dowdier cousin of impulse’, to ‘[get] inside another person’s state of heart or mind’.21 In asking audiences to channel their initial empathetic responses to source material, we aim to both bring them closer to the traces of the past, and replicate in them an integral part of our own experiences as researchers. This concept of an *effortful* empathy dependent on *historicised* understanding is integral to how we approach public engagement activities. The case studies that follow demonstrate our attempts to foster this response, and we return to the question of how and why this is important in the conclusion.

### Activity 1: Patient narratives, oral history and empathy (Tracey Loughran)

In February 2020, I designed and ran a session on ‘Patient Narratives, Oral History, and Empathy’ as part of an optional module on historical and contemporary contexts of medical education on an intercalated BSc in Medical Education. I was interested in this topic for several reasons. Some years earlier, one of my students had recalled an incident in which a young friend, recently diagnosed with an illness that had a high mortality rate, was tested for HIV prior to treatment. She overheard a doctor express hope that the test was positive, as he had never seen this particular illness interact with HIV before. I could rationalise the doctor’s response (just) as stemming from the desire to understand illness and so help patients, but saw making this comment within earshot of a patient as demonstrating low empathy. In fact, it was most likely simple carelessness, but, when later editing a chapter on whether reading literature increases empathy in health professionals, prehealth professionals and students outside of medical care, I often recalled this story.22 Later again, I noticed that for some interviewees on the Body, Self and Family project, an apparent lack of empathy among health professionals played an important part in their sense of pain and confusion following traumatic medical events. I wondered whether such stories could be redeployed, in a fashion that made reparation for these experiences, as part of medical students’ education in empathy during the clinical encounter.

The session was attended by almost all students on the module. It started with a short talk on empathy and patient narrative, and a discussion of preset reading from Arthur Kleinman’s *The Illness Narratives*, which argues that empathetically interpreting the patient’s narrative is essential to understanding the meaning of illness in the patient’s life, and to practising medicine humanely.23 This part of the session ensured that students were familiar with key concepts such as empathy and patient narrative, and allowed us to build up a rapport before moving onto potentially more challenging material. For the remainder of the session, we explored an oral history interview I had conducted with Janet (a pseudonym)24 in 2018 in three different formats: the interview summary (a concise guide to the interview, designed to help users identify parts relevant to their own research), a transcribed excerpt and an extract of the recording.

I chose this interview and excerpt partly for practical reasons: the session dealt with some complex concepts, and it was essential to allow sufficient time for students to engage with the material alone, and to discuss it as a group. It was also important to use an excerpt that clearly illustrated the dynamics of the doctor-patient relationship, and the effects of a specific medical encounter on the patient. In addition, I wanted the students to reflect critically on the role of the person who elicits a narrative (in patient case notes, the doctor; in oral history testimony, the interviewer). It therefore seemed fair to use an interview I had conducted myself. Janet’s interview met all these criteria. However, I also chose this excerpt because Janet’s narrative of a traumatic medical encounter (outlined below) had powerfully affected me during the interview, and I had often thought about it afterwards. I felt most confident that I could encourage students to think about empathy in relation to a narrative that had unquestionably provoked a strong emotional response in me.

The students had not seen any of the material relating to the interview prior to the session, and I introduced each format in successive stages. I asked questions to guide their reading/listening at each stage, and followed their own immersion in the material with group discussion. In introducing the same material in different formats, I hoped students would gain awareness of how the presentation of information in different ways can reveal or conceal different aspects of experience, and that they would reflect on how empathy is created, communicated and maintained.

When reading the interview summary, students were asked to consider what it revealed about Janet’s medical history, her encounters with health professionals, her self-understanding as a patient and whether the summary lacked any of the information necessary to interpret her story. This exercise approximated how health professionals might try to make sense of narratives they have not personally elicited. Despite dutiful reading, the students seemed unengaged with the dry, flat tone of the summary, which describes incidents in the interviewee’s life without attributing special weight or emotion to any of them. Most identified some of Janet’s medical encounters, but none picked out the episode she had narrated with most emotion during the interview.

Next, the students read a transcribed extract from the interview of around 2000 words. Here, Janet recounted her experience of pregnancy loss, including multiple instances when health professionals appeared to lack empathy towards her. I asked the students to note anything interesting or unusual about the form of the transcript, any points where they empathised with Janet, and any points where they felt other emotions. All students commented on the form of the transcription, which includes hesitations, pauses, laughter and interruptions, in the attempt to replicate the pattern of speech and flow of conversation.25 Reading transcriptions of this kind can be very jarring for those who have not previously encountered the form, but it forces the reader to slow down and pay attention to form as well as content. They noted empathy for Janet at the points I had anticipated, and some found her story distressing. Unexpectedly, their empathy for Janet quickly turned to open discussion about their own fears of failing to listen to patients, particularly given the time constraints of their usual clinical encounters.

Finally, we listened to a recording of the extract they had just read. I asked the students to consider whether Janet’s voice or speech sounded different to how they had expected; if she sounded emotional at certain points of the recording, and to identify those emotions; and how the interviewer (me) might have responded differently in any place. After listening to the recording, the students all remarked on Janet’s accent, which was clearly identifiable as belonging to a particular English region. Most (but not all) students felt that hearing Janet’s accent and the rhythm of her speech provided further clues to her personality. They also re-interpreted Janet’s response when she made a formal complaint that was ignored. They *read* the emotion in this part of the interview as sadness, but they *heard* it as fury. This prompted another unexpected discussion, on how they might listen for pauses, emphases, repetition and changes of tone in their own clinical encounters. This aspect of the session supports Janet Weston’s contention that ‘something very particular can be lost when oral histories are expressed and analysed in written form: the gestures, the performance, the tone and emotional content, the exchange between interviewer and interviewee, the voice itself’, and emphasises the value of putting audiences in touch with the raw materials of history.26 I also shared my own feelings that as an interviewer, I had handled some aspects of the interview badly, but reassured them that it is possible to make amends for these mistakes by careful listening and attention to body language of the interviewee/patient.

In retrospect, when I initially designed this session, I did not pay sufficient attention to the challenges health professionals face in an underfunded system. As a result of conversations with the module convenors, as well as the session itself, I became aware that medical students usually did not lack empathy, but rather time, resources and knowledge about how to ‘read’ and communicate empathy in the clinical encounter. In addition, as Archer and Greenlees show in their commentary in this volume, medical students may also be struggling with feelings of guilt or shame about their own role as learners in situations where individuals required care. My initial approach to the session did not consider the realities of these students’ experiences, and ignorance borders arrogance. In the months since I ran this session, the COVID-19 epidemic has led to renewed public discourse on the structural constraints under which health, medical and caring personnel operate, and this context has further challenged my assumptions.

Despite flaws in my initial assumptions, postsession feedback showed that students had benefited from the session. They identified as ‘take home’ messages the difficulty of predicting how a patient’s narrative might develop; the need to adapt their own style of communication in response to the patient’s emotional expression; the importance of not unthinkingly deploying medical jargon in response to human tragedies; and the necessity of both listening sensitively, and allowing the patient space to speak. Although the students already felt empathy for patients, the oral historian’s skills in listening and interpretation helped sensitise them to *how* emotion is expressed in patient narratives, and suggested how empathy might be actively communicated in the clinical encounter. The appropriate means of expression is something they must work out for themselves as their careers develop; as Rosie Harrison’s discussion elsewhere in this special issue shows, professional detachment can be necessary to manage emotion *so that* an appropriate kind of caring relationship can be maintained. However, this session might provide them with a greater range of tools to build a communication style that suits them and meets both their needs, and those of their patients. Meanwhile, the session prompted me to engage with the extensive literature on ‘compassion fatigue’, as well as popular medical memoirs that explore this phenomenon, so that I could run similar sessions in future with greater sensitivity to the working lives of participants.27 A new awareness of ‘profound differences in perspectives’ between historians and practitioners therefore provides the tools to start bridging that gap in the pursuit of ‘meaningfully engaged’ activities.28

### Activity 2: What do everyday health objects sound like? (Kate Mahoney)

In February 2020, the Body, Self and Family team participated in a ‘Valentine’s Late’ event. Led by the University of Roehampton’s Surgery & Emotion project, the event aimed to ‘explore the rich feelings…from compassion and romance, to anxiety and fear’ associated with health encounters.29 Museum and library ‘lates’ are typically promoted to younger adult audiences as an opportunity to view exhibitions outside normal opening hours in a friendly, social space.30 They incorporate talks, workshops, and food and drink. This particular Late was organised in collaboration with the Royal College of Nursing (RCN) Library and Archive, which promotes itself as supporting RCN members to ‘develop professionally and explore nursing and its history’.31 The event therefore attracted a mixed audience. Participants included young adults who were studying nursing and the history of medicine, many of whom attended with their friends; medical professionals; academic historians; and members of the public, young and old, who were interested in nursing and its history.

For the event, I created the activity ‘What Do Everyday Health Objects Sound Like?’, which aligned oral history recordings with historical objects to encourage participants to think about how they might define an ‘everyday health object’. The activity reflects the holistic construction of health we have championed on the Body, Self and Family project. The event’s open atmosphere, however, also engendered discussion on how the participants felt when they listened to the interviews. These exchanges illuminated the various ways in which participants built empathetic connections with the women’s voices featured in the activity. These differing formulations of empathy also prompted my own reflections on its role in the oral history interviews I had conducted for the project. The activity therefore reiterated how engagement activities enable historians to develop invaluable two-way conversations about history, empathy and identification across a range of public audiences.

In developing the activity, I located references to everyday health objects within the Body, Self and Family oral history interviews. One interviewee, born in 1944, cited the significance of a National Dried Milk tin in her childhood memories of health—her mother stored the family first aid kit in a repurposed tin.32 Another interviewee, born in 1942, worked in the shop on her parents’ Canvey Island campsite.33 She described how she served menstrual products to customers in black plastic shopping bags—a process that she felt promoted periods as ‘something horrible’.34 A further interviewee, born in Cardiff in 1966, described the lack of facilities at her primary school when she started her periods.35 She successfully campaigned for her daughters’ primary school to install sanitary bins to ensure they did not endure the same experience.36 In the selected extracts, the objects discussed by the interviewees did not immediately have medical connotations, serving instead as a container for foodstuffs, a receptacle for waste and a carrier for shopping items. By reflecting on their own experiences, however, the interviewees imbued the objects with purposes, emotions and values that rendered them intrinsic to the women’s sense of their everyday health. On the Body, Self and Family project, we explore the expansive ways in which women comprehended their health across their day-to-day lives. I felt that the interviewees’ discussion of everyday objects in the context of their health effectively showcased this aim.

The RCN activity drew on these oral history accounts to bring the objects cited by the interviewees to life. I placed MP3 players containing the interview extracts inside a National Dried Milk tin, a black shopping bag and a small bin, and plugged headphones directly into each object. I wanted to understand if situating women’s voices within the objects enhanced the participants’ listening experience; perhaps introducing an additional tactility or enabling listeners to visualise objects as they were discussed. I thought these factors could lend further tangibility to the events and experiences that interviewees recalled, therefore augmenting the participants’ empathetic engagement with the women’s everyday health experiences.

Multiple participants who engaged in this activity cited an empathetic response to the women’s voices due to their personal identification with the experiences recounted. In her discussion on the psychosocial benefits of oral storytelling, Hibbin asserts that the oral narration of experiences builds empathy because it enhances a ‘double-minded’ understanding of one’s self and others.37 The experiences of listening to oral history recordings and engaging with historical objects, appeared to engender comparable ‘double-minded’ understandings of women’s everyday health experiences in the RCN Late attendees; understandings based on both their own experiences and a desire to understand the women whose voices they heard. These empathetic responses based on personal identification took several forms.

A participant who was also a medical practitioner noted the value in listening to a series of women’s health experiences that were clearly positioned in the past. When working, the practitioner was obliged to respond to patients’ symptoms as they were being experienced. The contemporary immediacy of this exchange meant that the practitioner did not necessarily envisage the medical encounter as an event that the patient would later remember and reflect on. By engaging with a historical health narrative, the practitioner was alleviated of the pressure to immediately respond and provide a practical solution. As a result, they were attributed the mental and emotional space to develop an increasingly empathetic and reflective connection with the interviewee, while concurrently learning more about the long-term impact that everyday health experiences had on women’s lives.

One participant also stated that listening to the recordings and handling the objects ‘took me right back’. Her statement implied that she belonged to the same generation as the women interviewed and therefore felt an empathetic connection because she shared comparable experiences with the interviewees. For some attendees, this personal identification via shared experience spanned divergent national and cultural contexts. One participant—responding to the interviewee who recalled concealing menstrual products in black bags—described encountering comparable practices while growing up in India. In recalling her own experiences, the participant expressed identification with both the account on the recording and the object itself, which she picked up and held with a sense of familiarity. Wilton details how including objects in oral history interviews helps to capture specific aspects of an individual’s narrative. This process imbues the object with its own story, therefore generating new understandings of its significance within the interviewee’s life.38 In using the shopping bag as a prop, this participant reiterated the significance of the object within her own life, while also lending tangibility to her empathetic connection with our oral history interviewee.

Other attendees acknowledged intergenerational identification with the women’s voices. Their intergenerational empathy arose from reflections on similarities and differences between their experiences and those of older women. However, their empathy was also oriented around the sense that the women’s voices could provide insight into the lives and experiences of their own family members—the everyday health encounters of their mothers and grandmothers. This familial expression of empathy appeared to replicate the intergenerational framework within which several of my oral history interviews for the project took place. When conducting these interviews, I was surprised at the extent to which my empathetic connection with the interviewees was based on the fact that they were the same age as my mother. Interviewees also expressed empathy for my thoughts and feelings because they assumed I was the same age as their children, and therefore might share comparable experiences with them. As Roper and Duffett note, researchers exploring history and public engagement routinely find that ‘people are more likely to feel a sense of connection to the past through their family’.39 This research references families as a trusted source of historical knowledge. However, as indicated at the RCN Late and through my own interviews, in both research and public engagement settings, individuals might adopt a familial framework to understand and empathise with past experiences.

Another participant connected her empathetic response to the vocal tremors she could hear on the recordings. She felt that these tremors demonstrated how recalling memories was itself an emotional experience for the interviewees. Her empathetic connection with the interviewees was built on both an understanding of the emotion connected with past experiences, and the emotion associated with its memory ‘even after all this time’. The participant’s response highlights the value of actively encouraging listening as a means to elicit empathy for women’s voices in disseminating them to public audiences. Historians have also emphasised the importance of listening to oral history recordings when reusing interviews in research, rather than simply drawing on transcripts. Gallwey describes how listening to interviews from the British Library’s ‘Millennium Memory Bank’ project drew her into a close relationship with its contributors: ‘I felt very attuned to individual characters, their voices and ways of speaking and to the uniqueness of their stories’.40 Karpf argues that the voice is a ‘rich medium in its own right’.41 She states that the voice requires a historian’s ‘instinctual response which belongs more usually in interpersonal relationships than in traditional scholarship’.42 At the RCN Late, the act of listening personalised the women’s voices. As Godfrey highlights, the empathetic relationship between the historian and participant is invaluable to both the oral history interview and any subsequent analysis.43 In listening to oral history accounts, our public engagement participants also developed an interpersonal relationship with the project’s interviewees, based in part on identification with the women’s emotional vocalisation of particular experiences; we might draw a connection here with Slobogin’s argument elsewhere in this issue that the humanising details in photographs can encourage stronger feelings of empathy by providing access to emotions that are not easily or immediately articulable, or not necessarily articulated in different contexts.

When staging the activity, I included transcripts alongside recordings to ensure accessibility. One attendee, however, chose to read the transcript, stating that they did not like listening. I was surprised at how strongly I felt that this action disrupted the value of the activity. This feeling belied my assumption that the vocalisation of women’s experiences was intrinsic to the activity’s capacity to elicit empathy. In acknowledging this assumption, I also became aware of the significance I attribute to listening effectively in my own oral history practice. My empathy for oral history interviewees is based on both listening to the experiences that they recall, and seeking to understand how the process of remembering makes them feel during the interview process.

During the RCN event, several participants expressed surprise that the oral history recordings contained women’s voices as opposed to the mechanised ‘bleeps’ that they associated with medical objects. ‘It’s not making a sound’, one participant stated, ‘It’s just talking’. In this context, the vocalisation of women’s experiences did not always stimulate an empathetic response among event attendees. My ambition to use oral history recordings to disrupt assumptions about everyday health objects occasionally generated confusion that stymied participants’ capacity to empathise with the women’s voices. This demonstrates the potential for different aims to conflict with each other in public engagement activities.

### Activity 3: Could you be an agony aunt? (Daisy Payling)

At the RCN Late, I ran an adapted version of a third public engagement activity drawing on women’s voices. ‘Could You Be an Agony Aunt?’ uses letters and responses from problem pages in 1970s magazines for teenagers and adult women. Women’s magazines have been sites of advice and support for women for centuries.44 The letter-response format was integral to fostering a ‘supportive community’ within and beyond the magazine’s pages.45 Agony aunts, as emotional advisors, played an especially important role in creating this ‘fiction of friendship and trusted relationship’.46 Crucially, however, agony aunts fulfilled a dual role: they offered both ‘serious emotional advice and voyeuristic entertainment’.47 ‘Could You Be an Agony Aunt?’ embraces the dual role of problem pages to provoke empathetic responses in participants to these voices from the past.

Of course, letters to agony aunts only provide brief snapshots of past lives and experiences. Moreover, the voices of girls and women do not appear unmediated in this form. The letters on magazine problem pages were heavily edited. Virginia Ironside, agony aunt for *Woman* in the late 1970s, claimed that agony aunts ‘are writers first and foremost’, maintaining that she was employed for her ‘writing and editorial skills rather than for her caring and compassion’.48 Nevertheless, agony aunts attempted to retain the individual voices and personalities of readers as they edited the letters down to fit the page.49 As Claire Langhamer argues, the ‘interplay of individual self-knowledge and expert advice’ in printed letters and responses can still offer a window into past subjectivities.50

The premise of ‘Could You Be an Agony Aunt?’ is simple. The activity reproduces questions and answers from problem pages, but presents them separately from each other. Questions are scattered on one half of a table and responses on the other half. Participants must match questions with responses. Some responses could plausibly match more than one problem, so participants have to be aware of tone and content—to a certain extent imagining themselves as 1970s agony aunts. During and after the activity, I ask follow-up questions, including ‘What do you think of this advice?’, ‘What advice would you give to this person if they were your friend?’, ‘Do these problems resonate with you?’ and ‘How do you think teenagers or women’s lives have changed since the 1970s?’. I have run the activity at several events, tailoring it to diverse audiences. The first iteration of the activity was developed for school students aged 14–15 years as part of the University of Essex’s Digital Arts Festival. For this event, I selected five problems representing areas including body image, confidence, sexuality (including homosexuality) and relationships, and made a worksheet instructing participants to draw a line linking the problem to the correct response. These were areas of concern raised by teenage letter writers to magazines, but are also areas that the PSHE Association draws attention to in its current guidelines.51 For stands at public history events with LGBTQ+ audiences and at the RCN Late, I created the tabletop version described above. The LGBTQ+ events stimulated the inclusion of more questions on homosexuality, and for the 16+ audience at the RCN Late I used problems and responses from *She* magazine’s sex column.

In developing the activity, I had to select and edit letters and responses, taking into account space constraints and the need to avoid overburdening participants with text. In doing this, I replicated the role of the agony aunt compiling the problem page. I empathised with the experience of Nick Fisher, agony uncle for *Just 17* in the 1990s, of ‘looking at your page and thinking, “I can't have five letters all about this, so I've got to have a bit of this, a bit of that”’.52 Like many agony aunts, however, I tried to accurately represent the voices in the letters. Also, as with agony aunts, careful selection and editing was necessary to create an activity that would ‘hook’ participants. As Suzie Hayman, former agony aunt for *Woman’s Own*, explains: ‘As an agony aunt, I think I owe my readers professionalism and empathy and knowledge and understanding… But I also owe them entertainment, because they're not gonna read if it’s just a professional screed’.53 With questions on homosexual feelings, weight loss, spots, feeling apprehensive about going out with friends, and how to get better at walking in high-heeled shoes, I wanted the problems to balance relatability with light-heartedness. These problems were also selected on the merits of their matching advice. Advice needed to be sensitive and sensible, even accounting for changing attitudes over time, so as not to do harm. In particular, the weight loss question and answer pair was selected because it offered no specific suggestions on how to lose weight.

‘Could You Be an Agony Aunt?’ was therefore designed to create empathy with past experiences, and to entertain in the present. The magazine quiz-style title immediately challenges participants to place themselves in the agony aunt’s shoes: an empathetic act and an empathetic persona. Magazine quizzes encourage participants to imagine new selves and can direct the nature those selves take.54 In this activity, the new imagined self is an agony aunt, but the quiz-style title also transports adult participants to the playful spaces of their teenage years, with the challenge of the task reinforcing the sense of play for participants of all ages. It draws on the recognised role of play and creative engagement in supporting learning.55 In asking participants to actively imagine themselves as agony aunts, the activity also follows Jamison’s conception of empathy as effortful. Participants do not simply empathise with letter writers, but need to pay attention both in identifying the real-life advice, and considering the follow-up questions that require reflection on the effectiveness of the advice, and other potential responses. These questions consolidate the empathetic engagement, offering opportunities to reflect on how advice might change and how people might deal with problems in the present.56

In practice, school students were able to identify with the problems and with the advice. They successfully put themselves into the position of agony aunt to complete the activity and extended their empathetic engagement to suggest their own advice. A problem from a 13-year-old who felt happier staying at home with their parents than going out with their friends seemed to encourage a particularly empathetic response, but perhaps this was one that the school students, a few years older than the question writer, felt some distance from, enabling them to talk about it more openly in front of their peers.

Adult participants at the RCN Late engaged with the task in a slightly different way. The inclusion of sex problems from *She* magazine amped up the entertainment aspect of the task. It drew participants into reading the problem page as a voyeuristic experience, described by Nick Fisher as the ‘slightly guilty pleasure, of reading your big sister’s magazine that you shouldn't really be reading… because it’s given you some kind of Peeping Tom-like experience into human frailty’.57 However, some participants’ surprise at the sexual practices described in letters led them to express their own uncertainty about the matters in question, while others empathised not only with the voices of past readers, but with their own past selves. Although many participants were healthcare practitioners, they tended to relate this task to their own experiences rather than those of their patients. Sympathetic responses to young readers questioning their sexuality in the 1970s prompted spontaneous reflections on how Section 28^58^ had affected participants’ own adolescent understanding of sexuality, and how they would have welcomed an empathetic ear. Some also expressed surprise at agony aunts’ nuanced responses to these questions. In creating new empathetic connections with the agony aunt, the quiz therefore challenged popular stereotypes of agony aunts as condescending and conservative, and created a new ‘horizon of context’ for these participants.59

## Conclusion: empathetic afterlives?

Collectively and apart, we have now conducted multiple engagement activities, with many different audiences. We have refined and adapted activities in response to the feedback of participants, and solicited feedback from academics and others with experience of engaging diverse audiences. We believe these activities are valuable in introducing audiences to women’s voices and experiences of ‘everyday health’, and in stimulating reflection on past and present capacities for action. The participatory elements of these activities are crucial in engaging the attention of those who take part, and provoking an effortful empathy that might resonate beyond the time span of each event. It remains challenging, however, to create activities and environments that foster the *historicised* empathy that is our ideal. One of the lesser problems is where to pitch information, and how much context to provide, for audiences with differing levels of background knowledge and of different ages. Another issue is how to ensure that our activities, which foreground women’s voices, feel relevant to mixed-gender audiences. We have found that mixed-gender adult audiences are usually receptive, but activities with schools run the risk of marginalising male pupils by not including the ‘everyday’ experiences of boys and men in the past, or accidentally reinforcing the notion that femininity is the ‘problem’ that needs to be solved.

Most troubling is the issue of representation.60 For most of our period, mass cultural productions either exoticised or rendered invisible BAME and LGBTQ+ women. Our activities need to portray how racism and heterosexism structured the ‘everyday health’ experiences of these women, through unthinking exclusion as well as documentable oppression. But how do you represent and historically contextualise invisibility, especially when time is short and audiences do not want a lesson? Equally, introducing BAME and LGBTQ+ voices primarily to illustrate experiences of racism and heterosexism risks reducing these women’s multifaceted lives to oppression and victimhood. As one of our interviewees sighed when describing a heterosexist medical encounter, ‘you get tired, I think of always being part of somebody else’s education’.61 We are particularly keen not to represent BAME and LGBTQ+ women’s experiences in primarily negative terms to adolescents. As they struggle with racism and heterosexism in their own lives, they need opportunities to empathise with the successes, pleasures and mundanities of the lives of past women with whom they share aspects of identity—the opportunities that white, heterosexual girls and women can take for granted.

These are complicated questions, but they are vital to stimulate historicised empathy. It is easy to generate superficial empathy by treating the past like a pick ‘n’ mix of treats for our selection and consumption. As Jenny Crane shows in her thoughtful and thought-provoking exploration of public engagement as a method in social histories of medicine, it is much more difficult to guide audiences towards a sense of the messiness and irresolvability of history—but it is also our responsibility as historians.62 Because we usually do not know in advance who will be in each audience, and what aspects of their identity and experience they may bring to bear on interpreting these activities, we cannot select materials that we think are likely to resonate especially with particular groups. Instead, we try to ensure that issues of visibility and invisibility are threaded through our materials and design of activities. This is partly a matter of positive representation (images of BAME women, problem page letters from LGBTQ+ adolescents), but it is also a matter of inviting reflection on what is not said or not visible. Some audience members will be immediately receptive to these questions, while others may not even notice that they have been implicitly or explicitly raised—but we can only open up possibilities for potential responses, not determine the nature of those responses.

These difficulties are sharpened when we consider how to replicate some of the activities we have developed in forms that do not depend on our active presence in the room. What is lost, and are there any potential gains? Our first case study, the activity with medical students, is most like a conventional seminar. It will be simple to prepare the materials in a format that allows a tutor in a medical school to run the session, including a historical crib sheet. The experience of the oral historian is lost, and the productive clash of different disciplinary approaches. However, these losses could be balanced if the session is run by a clinician-educator who understands and anticipates the challenges that medical students face.

The agony aunt quiz already exists in two forms which allow participants to engage with the activity remotely. We adapted the worksheet from the Digital Arts Festival so that school students could work through it individually. We included historical context and discussion questions, and reduced the number of problem/answer pairs from five to three in order to include a section prompting students to answer one of the problems themselves. Feedback from teenage participants suggested that this adaption was not wholly successful. Two participants described the reduced matching element as ‘too simplified’. Both wanted more opportunities to embody the agony aunt; to create and answer problems from their own perspectives, and in the persona of a 1970s agony aunt. However, another participant stated that they felt ‘uncomfortable’ offering advice, despite not having to share this with anyone. This participant also thought that there should be more space in the activity to challenge the agony aunt’s advice. This is complex feedback which we will work through as we redevelop the activity, but it illustrates the difficulties of replicating the task without one of us in the room to facilitate sensitive discussion of the problem/answer pairs as acute emotional objects, and critical engagement with them as historical sources.

We also reformulated the agony aunt activity into a digital quiz which we shared on Twitter.63 In digital form, each question has only two (both plausible) responses for participants to choose from. Once the participant chooses an answer, the next question automatically appears. A competitive element is added as participants score points for correct answers. This version of the quiz prioritises individual engagement and quick responses rather than discussion and thoughtful reflection. Choosing between two similar answers forces the participant to weigh answers up against each other looking for clues, rather than thinking about the context of the advice. Described as ‘fun’ and challenging, participants nevertheless had no opportunity to become immersed in the breadth of past advice or to stumble across repeated themes, and so the benefits of exploratory play were lost.64 This diminishes both the historical purpose and the potential for empathetic engagement that the activity offers. To combat some of these losses, in future iterations the digital activity will include historical context and questions prompting reflection. As demonstrated with the worksheet, however, written prompts cannot be relied on to replicate the face-to-face experience. The online version of ‘Could You Be an Agony Aunt?’ may remain pure entertainment for many users: but in some ways, this reflects the original form of the agony aunt page in magazines.

In the activity with everyday health objects, technological refinements could increase the participant’s agency and remove the need for the historian’s presence. Integrating speakers into objects, and installing a microcontroller that is programmed to start playing the recording when a participant interacts with the object, means participants can listen to the recording from the start, and without the barrier of headphones. An information sheet that explains the activity, alongside a feedback box for reflections, could partially replace conversations about the objects and histories. This removes the awkwardness that some participants may have felt about being watched while they listened to the recordings, and any obligation they felt to share their thoughts and feelings. Making it possible for participants to engage with the objects and recordings on their own terms could bolster quiet reflection and considered affective responses.

This is, perhaps, a good point on which to end our contribution to this special issue on healthcare, policy and the emotions. The majority of contributions to this collection consider how an emotions-based approach to the health humanities can inform policy-making and practice. Our own public engagement activities to date have been exploratory rather than seeking to achieve specific ‘impacts’. While recognising the potentially detrimental effects of instrumentalist approaches to ‘impact’,65 we also believe that the ‘impact’ agenda in UK Higher Education has had positive effects in encouraging many historians to work with different audiences, and (crucially) providing funding and institutional recognition for those who have always seen this as part of their work.66 At the same time, we are fortunate in that our funder chooses to emphasise ‘public engagement’ rather than ‘impact’. We have had the space and time to experiment with different kinds of activities, rather than feeling the pressure to achieve distinct ‘outcomes’. This freedom is carried into our interactions with audiences. If our aim is to encourage historical and historicised empathy through rendering both past and present less familiar, then the aftershocks of our activities should ripple through participants’ thoughts and feelings almost imperceptibly over time. Allowing space for slow and untraceable effects is, perhaps, another way to acknowledge the complexity and contingency of causality—a genuinely historical approach if ever there was one.

## Data Availability

No data are available.

## Notes

1. ”Body, Self and Family: Women’s Psychological, Emotional and Bodily Health in Britain, c. 1960–1990.” Wellcome Trust Investigator Award in the Humanities and Social Sciences. This research was funded in whole, or in part, by the Wellcome Trust [*Grant No.: 208080/Z/17/Z*]. For the purpose of open access, the author has applied a CC BY public copyright licence to any Author Accepted Manuscript version arising from this submission.

2. Stephen Hinchliffe *et al.* (2018), “Healthy Publics: Enabling Cultures and Environments for Health,” *Palgrave Communications*, 4, issue 57, 2.

3. Penny Summerfield (2004), “Culture and Composure: Creating Narratives of the Gendered Self in Oral History Interviews,” *Cultural and Social History*, 1, no. 1.

4. Cynthia White (1968), *Women’s Magazines, 1693–1968* (London: Michael Joseph), 155–78, 216–42.

5. Ros Ballaster et al. (1991), *Women’s Worlds: Ideology, Femininity and the Woman’s Magazine* (New York: NYU Press), 111.

6. Claire Langhamer (2018), “Who the hell are ordinary people?” Ordinariness as a Category of Historical Analysis* , Transactions of the Royal Historical Society*, 28.

7. Tracey Loughran (2020) ““The most helpful friends in the world”: Letters Pages, Expertise and Emotion in British Women’s Magazines, c. 1960–1990,” in *Women’s Periodicals and Print Culture in Britain, 1940s–2000s: The Postwar and Contemporary Period*, eds. Laurel Forster and Joanne Hollows (Edinburgh: Edinburgh University Press).

8. Kadija Sesay (2002), “Publishing, Newspapers and Magazines,” in *Companion to Contemporary Black British Culture*, ed. Alison O’Donnell (London and New York: Routledge): 250–53.

9. Michael Roper (2005), “Slipping Out of View: Subjectivity and Emotion in Gender History,” *History Workshop Journal*, 59 no. 1.

10. Carolyn Faulder (1977), “Women’s Magazines,” in *Is This Your Life? Images of Women in the Media*, eds. Josephine King and Mary Stott (London: Virago): 173–94; Beatrix Campbell and Anna Coote (1982), *Sweet Freedom: The Struggle for Women’s Liberation* (London: Picador), 189–210.

11. Janice Winship (1987), *Inside Women’s Magazines* (London: Pandora Books); Ros Ballaster et al. (1991), *Women’s Worlds*; Angela McRobbie (1991), *Feminism and Youth Culture: From Jackie to Just Seventeen* (London: Macmillan).

12. Lynn Abrams (2010), *Oral History Theory* (London and New York: Routledge), 54–105.

13. With thanks to Alison Twells for this insight.

14. Paul Bloom (2016), *Against Empathy: The Case for Rational Compassion* (London: Vintage), 15.

15. Bloom, *Against Empathy*, 5.

16. Keith Crawford (1995), “A History of the Right: The Battle for Control of National Curriculum History, 1989–1994,” *British Journal of Educational Studies*, 43: 441.

17. Peter Clements (1996), “Historical Empathy – R.I.P.?,” *Teaching History*, 85: 6–8.

18. Qualifications and Curriculum Authority, *History: Programme of Study for Key Stage 3 and Attainment Target* (2007), 112. Accessed April 26, 2020, http://archive.teachfind.com/qcda/curriculum.qcda.gov.uk/uploads/QCA-07-3335-p_History3_tcm8-189.pdf

19. Janet Weston’s account of using oral history to create an audio drama similarly emphasises the role of imagination and creativity in historical work. See Janet Weston (2019), “Oral Histories, Public Engagement and the Making of Positive in Prison,” *History Workshop Journal*, 87: 211.

20. Notable exceptions include Carolyn Steedman, *Dust* (Manchester: Manchester University Press, 2002); Emily Robinson (2010), “Touching the Void: Affective History and the Impossible,” *Rethinking History*, 14, no. 4 (2010); Michael Roper (2014), “The Unconscious Work of History,” *Cultural and Social History*, 11, no. 2 (2014).

21. Leslie Jamison (2014), *The Empathy Exams: Essays* (London: Granta), 23.

22. Lauren Fowler and Sally Bishop Shigley (2018), “The Expectation of Empathy: Unpacking Our Epistemological Bags While Researching Empathy, Literature, and Neuroscience,” in *Emotion and the Researcher: Sites, Subjectivities and Relationships*, eds. Tracey Loughran and Dawn Mannay (Bingley: Emerald).

23. Arthur Kleinman (1988), *The Illness Narratives: Suffering, Healing and the Human Condition* (New York: Basic Books), xi–xv, 1–55.

24. "Janet" (2018). Interviewed by Tracey Loughran, August 6. All BSF interviewees are referred to by pseudonyms in this article.

25. Raphael Samuel (1971), “Perils of the Transcript,” *Oral History*, 1, no. 2.

26. Weston, “Oral Histories,” 219.

27. Fiona Cocker and Nerida Joss (2016), “Compassion Fatigue among Healthcare, Emergency and Community Service Workers: A Systematic Review,” *International Journal of Environmental Research and Public Health*, 13, no. 6 (June); Joanna Cannon (2019), *Breaking and Mending: A Junior Doctor’s Stories of Compassion and Burnout* (London: Profile Books); see also McQueen, Hammond Mobilio and Moulton in this special issue.

28. See Hinchcliffe *et al*, “Healthy Publics,” 7.

29. Royal College of Nursing Library and Archives and ‘Surgery & Emotion’, “Valentine’s Late: Open Hearts, Racing Pulses,” accessed April 26, 2020,

30. https://www.eventbrite.co.uk/e/valentines-late-open-hearts-racing-pulses-tickets-68256847067%23. Accessed April 21, 2021.

31. Anna Semmens and Teresa Doherty (2015), *RCN Library and Archive Services: Five Year Plan 2016–2020* (London: Royal College of Nursing), 2.

32. Abigail (2018). Interview with “Abigail,” January 25, 2018.

33. Hannah, 2018. Interview with “Hannah,” February 2, 2018.

34. Mahoney. Interview with “Hannah.”

35. Hattie, 2018, Interview with “Hattie,” May 17, 2018.

36. Mahoney. Interview with “Hattie.”

37. Rebecca Hibbin (2016), “The Psychosocial Benefits of Oral Storytelling in School: Developing Identity and Empathy Through Narrative,” *Pastoral Care in Education*, 34, no. 4: 224; Simon Baron-Cohen, *Zero Degrees of Empathy: A New Theory of Human Cruelty* (London: Penguin).

38. Janis Wilton (2009), “Belongings: Oral History, Objects and an Online Exhibition,” *Public History Review*, 16: 3.

39. Roper, Michael and Duffett, Rachel (2018), “Family Legacies in the Centenary: Motives for First World War Commemoration among British and German Descendants,” *History & Memory*, 30, no. 1 (Spring/Summer): 77.

40. April Gallwey (2013), “The Rewards of Using Archived Oral Histories in Research: The Case of the Millennium Memory Bank,” *Oral History*, 41, no. 1 (Spring): 44.

41. Anne Karpf (2014), “The Human Voice and the Texture of Experience,” *Oral History*, 42, no. 2 (Autumn): 50.

42. Karpf, “The Human Voice,” 54.

43. Barry S Godfrey (2004), “Self-Identity, Empathy and Sympathy in Reading Transcripts,” in *Narrative, Memory and Identity: Theoretical and Methodological Issues*, eds. David Robinson et al (Huddersfield: University of Huddersfield): 59.

44. Laurel Forster (2015), *Magazine Movements: Women’s Culture, Feminisms and Media Form* (New York and London: Bloomsbury),158.

45. Fiona Hackney (2016), “Getting a Living, Getting a Life: Leonora Eyles, Employment and Agony, 1925–1930,” in *Women in Magazines: Research, Representation, Production and Consumption*, eds. Rachel Ritchie, Sue Hawkins, Nicola Phillips and S. Jay Kleinberg (New York and London: Routledge), 108.

46. Penny Morris (2004), “From Private to Public: Alba de Céspedes’ Agony Column in 1950s Italy,” *Modern Italy*, 9, no. 1: 12.

47. Claire Langhamer (2013), “Everyday Advice on Everyday Love: Romantic Expertise in Mid-twentieth Century Britain,” *L' homme: Zeitschrift für feministische Geschichtswissenschaft*, 24, no. 1: 43.

48. Virginia Ironside, *Problems! Problems! Confessions of an Agony Aunt* (London: Robson Books), 131.

49. Tracey Loughran. Interview with Suzie Hayman, January 7, 2019; Tracey Loughran. Interview with Irma Kurtz, September 4, 2018.

50. Langhamer, “Everyday Advice on Everyday Love,” 39.

51. PSHE Association, *Programme of Study for PSHE Education (Key Stages 1–5).* Accessed January 26, 2021 https://www.pshe-association.org.uk/curriculum-and-resources/resources/programme-study-pshe-education-key-stages-1%E2%80%935

52. Tracey Loughran. Interview with Nick Fisher, September 1, 2018.

53. Loughran. Interview with Suzie Hayman.

54. Elizabeth Wilson (1982), *Mirror Writing: An Autobiography* (London: Virago, 1982), 16; Ana Cristina Ostermann and Deborah Keller-Cohen (1998), “Good girls go to heaven; bad girls… learn to be good: Quizzes in American and Brazilian Teenage Girls’ Magazines,” *Discourse & Society*, 9, no. 4: 534.

55. Mitchel Resnick and Ken Robinson (2017), *Lifelong Kindergarten: Cultivating Creativity Through Projects, Passion, Peers, and Play* (Cambridge, MA: MIT Press).

56. Graham Gibbs (2015), “Much Learning is Acquired by Doing, but Seldom Only by Doing,” *SEDA*, 31: 2.

57. Loughran. Interview with Nick Fisher.

58. Section 28 stated that local authorities should ‘not intentionally promote homosexuality’ or ‘the teaching […] of the acceptability of homosexuality as a pretended family relationship’.

59. Jamison, *The Empathy Exams*, 23. An example of this popular caricature of agony aunts is Julia Davis and Vicki Pepperdine’s podcast “Joan and Jericha.” See Fiona Sturges (2019), “Joan and Jericha: the Derek and Clive of Agony Aunts,” *Financial Times*, October 6.

60. A related issue, which we do not have space to discuss in depth here, is our own *presentation* as individuals and as a team: when we interact with different publics, they know nothing more about our own identities (ethnicity, sexual orientation, social class and so on) than what appears to be visible, and *their* assessments of who *we* are undoubtedly shape these brief but charged intersubjective exchanges.

61. Daisy Payling, Interview with “Josie,” December 12, 2019.

62. Jennifer Crane (2020), “’The NHS … Should not be Condemned to the History Books’: Public Engagement as a Method in Social Histories of Medicine,” *Social History of Medicine*: 8–10, 16. Advance access, (May 15): https://doi.org/10.1093/shm/hkaa041.

63. You can play the game here: https://www.sporcle.com/games/BSF/could-you-be-a-1970s-agony-aunt-2.

64. Lee Skallerup Bessette (2013), “It’s Time to Play: Games, Gamification, and Active Learning,” *Hybrid Pedagogy*, March 25. Accessed April 28, 2020 https://hybridpedagogy.org/its-time-to-play-games-gamification-and-active-learning/.

65. Laura King and Gary Rivett (2015), “Engaging People in Making History: Impact, Public Engagement and the World Beyond the Campus,” *History Workshop Journal*, 80.

66. Matthew Grant (2017), “History and Policy,” in *A Practical Guide to Studying History: Skills and Approaches*, ed. Tracey Loughran (London: Bloomsbury), 239.
